# Localization and Functional Analysis of CtLTP8, an Extracellular Vesicle Protein That Enhances Resistance to *Botrytis cinerea* in Safflower

**DOI:** 10.3390/plants15101527

**Published:** 2026-05-16

**Authors:** Kang Ma, Yongmei Luo, Kangjun Fan, Xiaoyan Wang, Jiao Liu, Rui Qin, Zhaojun Wei, Hong Liu

**Affiliations:** 1 Hubei Provincial Key Laboratory for Protection and Application of Special Plant Germplasm in Wuling Area of China, College of Life Sciences, South-Central Minzu University, Wuhan 430074, China; mvp2003@126.com (K.M.); yongmeiluo5316@163.com (Y.L.);; 2 College of Biology and Food, Shangqiu Normal University, Shangqiu 476100, China; 3 School of Biological Science and Engineering, North Minzu University, Yinchuan 750021, China

**Keywords:** *Carthamus tinctorius*, biotic stress, lipid transfer proteins, plant immunity

## Abstract

Safflower (*Carthamus tinctorius* L.) is an economically important crop, yet its production is severely threatened by fungal diseases including *Botrytis cinerea*. The molecular mechanism underlying disease resistance in safflower remains largely unclear. Extracellular vesicles (EVs), as vital carriers for cross-kingdom communication and transport, play crucial roles in plant antifungal defense. Lipid transfer proteins (LTPs), members of the pathogenesis-related protein 14 family, have also been shown to be key players in plant disease resistance. The promising resistance-related candidate gene *CtLTP8* was previously identified via genome-wide association study (GWAS). In this study, a genome-wide analysis of the LTP gene family in safflower was performed. EVs were isolated from the apoplastic washing fluid of *B. cinerea*-infected safflower leaves, and proteomic analysis was performed. Numerous proteins associated with disease resistance, including CtLTP8, were detected by proteomic profiling. CtLTP8 was found to be present in EVs through molecular biological experiments. Moreover, stable overexpression of *CtLTP8* in safflower significantly increased resistance to *B. cinerea*. In summary, this study characterized the disease resistance-related proteome of safflower EVs, and verified the presence of CtLTP8 in EVs and its antifungal function, providing valuable gene resources and theoretical support for safflower disease-resistance breeding and research on EV-mediated plant immune mechanisms.

## 1. Introduction

*Carthamus tinctorius* L. (2*n* = 2*x* = 24) is an economically important crop belonging to the Asteraceae family. With strong stress tolerance to barren soil, drought, and heat, it is widely cultivated in arid and semi-arid regions of more than 60 countries around the world [1,2]. The economic value of safflower is reflected in the diversified utilization of its seeds and flowers. As an excellent oil resource, safflower seeds are rich in linoleic acid and are widely used as high-quality edible oil [3]. Safflower flowers are abundant in flavonoids, and hydroxy safflor yellow A, a unique active component, exhibits various significant pharmaceutical activities and is widely applied in medicines and functional foods. In addition, safflower is extensively used in industrial fields including dyes, feed, biodiesel, and cosmetics [4,5,6,7,8].

However, pathogen infection has become a critical bottleneck restricting the improvement in safflower yield and quality, among which fungal damage is particularly prominent. Foliar diseases easily break out on a large scale in the middle and late growth stages when rainfall occurs. Fungi such as *Botrytis cinerea* can induce typical foliar diseases [9,10,11]. At present, in-depth investigations into the molecular mechanisms of safflower disease resistance are urgently needed to provide high-value candidate genes for safflower disease-resistant breeding. Meanwhile, *B. cinerea* is also commonly used to study plant–fungus interactions [12,13].

Extracellular vesicles (EVs) are membranous vesicles secreted by prokaryotic and eukaryotic cells. Based on biogenesis mechanisms, EVs are classified into apoptotic bodies, microvesicles, and exosomes. EVs are capable of encapsulating bioactive substances such as nucleic acids, proteins, and lipids for intercellular delivery, and play vital roles in key biological processes including cell communication and immune responses [14,15,16]. Cross-kingdom substance transport mediated by EVs is demonstrated to exert core regulatory functions in plant–microbe interactions, and such critical interactions are mainly detected in the apoplastic microenvironment of plants [14,15,16].

Investigations of plant EVs were initiated with the first observation in carrot cells in 1967. Subsequent studies have verified that EVs are secreted by plant cells into intercellular spaces, and EVs are released by both plants and pathogens to mediate molecular communication between the two organisms [13,17,18]. Plant EVs originate from multivesicular bodies and exosome-positive organelles (EXPOs). EXPOs constitute a category of plant cell organelles/vesicles associated with unconventional protein secretion and marked by exocyst components. They have been documented to mediate the transport of cytosolic proteins to the cell surface and exhibit organellar properties distinct from those of canonical endocytic markers. Moreover, plant EVs carry bioactive cargoes such as RNAs and proteins and participate in pathogen defense responses [13,19,20,21]. EVs are secreted by both plant pathogenic fungi and oomycetes. Fungal EVs are loaded with virulence-related factors, while oomycete EVs are enriched in RxLR effector proteins. Pathogen-derived EVs are proven to enhance pathogenicity and trigger toxic responses in host plants. In addition, evidence has been provided that exogenous EVs can be taken up by both plant and fungal cells [22,23,24].

During plant resistance to fungal infection, plant EVs are demonstrated to exert critical antifungal effects by delivering functional proteins and nucleic acid molecules. Proteomic profiles of sunflower seedling EVs are shown to contain pathogenesis-related proteins such as PR4 and PR5, as well as defense-associated proteins including GDSL lipase. EVs secreted by sunflower seedlings are taken up by Sclerotinia sclerotiorum and suppress the growth of this fungus [20,25]. EVs derived from tomato root cells are loaded with proteins involved in plant–pathogen interactions, and are confirmed to inhibit spore germination of pathogenic fungi including *Fusarium oxysporum* and *B. cinerea* [26]. When infected by *Rhizoctonia solani*, rice plants are found to transport disease-resistant proteins such as OsJLL1 and OsDUF26 via EVs into fungal cells. Meanwhile, OsTET7, a marker protein of rice EVs, is known to possess independent disease-resistant functions [27]. The proteome of *Arabidopsis* EVs is also revealed to be enriched in numerous defense-related proteins, which are delivered as functional defensive molecules to counteract fungal infection [28]. Small RNAs (sRNAs) and AGO1 proteins are selectively loaded into plant EVs. In *Arabidopsis*, sRNAs are delivered via EVs into *B. cinerea* cells to silence target genes and attenuate fungal virulence [13,29]. Notably, mRNAs are reported to be transported by plant EVs into invading fungi and directly translated into functional proteins, leading to the inhibition of fungal growth [28]. Furthermore, recent studies have indicated that safflower EVs are taken up by *B. cinerea* and directly suppress the growth of this fungus [11].

Non-specific lipid transfer proteins (LTPs) are small cysteine-rich proteins widely distributed in plants, and are recognized as members of the pathogenesis-related protein 14 (PR14) family [30,31]. Four disulfide bonds formed by eight conserved cysteine residues, which contribute to a stable tertiary structure, and a central hydrophobic cavity capable of binding various lipids such as fatty acids and phospholipids are regarded as the core structural characteristics of LTPs. In addition, most LTP members are confirmed to contain an N-terminal signal peptide and are secreted into the extracellular space to perform diverse physiological functions [32,33,34].

LTPs are identified as multifunctional proteins involved in plant growth and development. Besides the core function in disease resistance, LTPs are widely demonstrated to participate in pollen tube adhesion and development, cuticular wax formation, seed development and germination, cell expansion, and other biological processes [35,36,37,38,39]. The expression of LTPs is induced by salt stress, drought, extreme temperature, and other adverse conditions, and plant tolerance is enhanced via the regulation of lipid metabolism or signal transduction [40,41,42,43,44,45].

In disease resistance, LTPs are shown to exert prominent effects against fungal diseases. Antifungal activities are directly achieved by disrupting the integrity of fungal cell membranes and inhibiting spore germination [46,47,48]. Indirect enhancement in antifungal capacity is mediated by regulating cuticular barrier function, participating in systemic acquired resistance (SAR) signaling, and mediating hormone pathways including jasmonic acid (JA) and abscisic acid (ABA) [46,47,48,49]. Meanwhile, LTPs are also involved in defense against other pathogens such as bacteria. A few LTP members are reported to negatively regulate immune responses by modulating programmed cell death to prevent excessive defense from inhibiting plant growth [50,51]. Given the vital roles of LTPs in plant antifungal immunity and multiple physiological processes, in-depth investigations into their molecular mechanisms are considered essential for understanding plant immune networks and crop disease-resistant breeding.

In this study, a disease resistance-related LTP gene in safflower was obtained via GWAS analysis, followed by genome-wide analysis of the safflower LTP gene family and proteomic identification of EVs from safflower leaves infected by *B. cinerea*. The presence of CtLTP8 in EVs was further identified, and its overexpression was confirmed to significantly improve resistance to *B. cinerea* mold in safflower. This study clarifies the presence of CtLTP8 in EVs and its function in enhancing resistance against *B. cinerea*, providing important gene resources and a theoretical basis for molecular breeding of disease-resistant safflower.

## 2. Results

### 2.1. LTP and LTP Gene Family

Previously published Genome-Wide Association Study (GWAS) data based on resequenced SNP loci of safflower populations associated with disease severity index were obtained from a previous study in this laboratory [11]. Reanalysis of these datasets was performed in the present study. A SNP locus located on chromosome 2 was found to be strongly associated with a gene encoding a lipid transfer protein.

Linkage Disequilibrium block (LD block) analysis was conducted for this SNP locus (Figure S1). The results indicated that a cluster of highly significant SNPs represented by Chr2_67754173 (*p* = 9.85 × 10^−7^) was located downstream of Chr2T10180100, a gene encoding an LTP protein. Thus, GWAS analysis suggested that Chr2T10180100 might serve as a high-value candidate gene for disease resistance in safflower.

**Figure 1:**
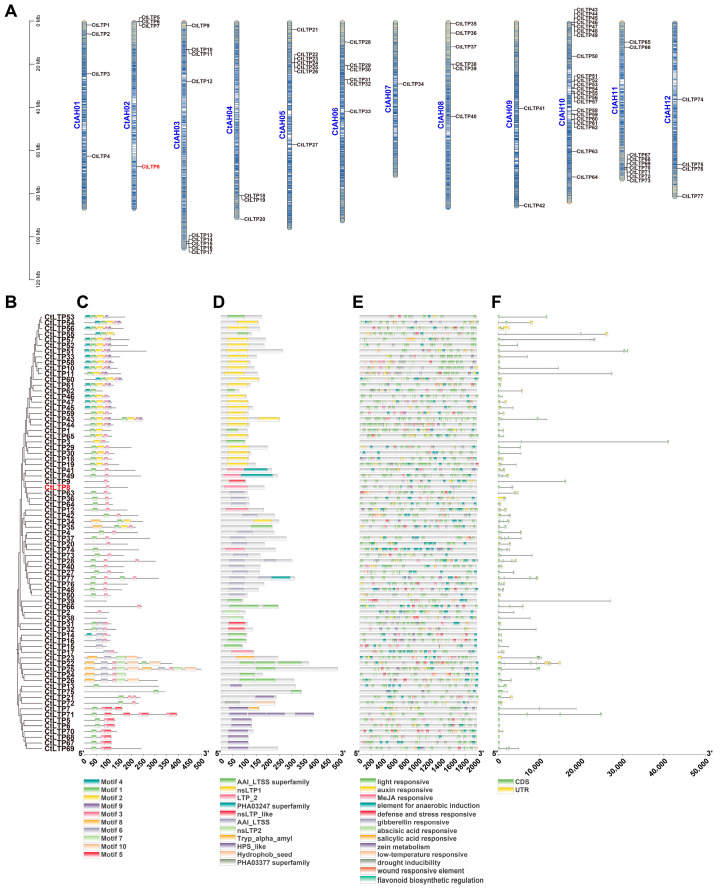
**The LTP gene family in safflower:** (**A**) chromosomal localization; (**B**) phylogenetic relationship; (**C**) motif analysis; (**D**) conserved domain analysis; (**E**) promoter analysis; (**F**) gene structure analysis.

Further, genome-wide analysis of the safflower LTP gene family was performed (Figure 1). Protein sequences from the whole safflower genome were analyzed using hidden Markov model (HMM) searches and BLASTP. Genes with truncated fragments or incomplete functional domains were excluded, and 77 members of the LTP gene family were finally identified.

The encoded amino acid sequences ranged from 77 to 497 amino acids (aa). After excluding extreme values, most LTP proteins measured 100–200 aa in length (e.g., 112 aa for CtLTP1 and 177 aa for CtLTP24) (Table S1). The 77 safflower LTP genes were designated LTP1 to LTP77 according to their chromosomal positions.

The 77 LTP family members were distributed across all 12 safflower chromosomes. Clustered distributions were detected on chromosomes 2, 3, 5, 8, and 11. Chromosome 10 contained the largest number of genes (22) and formed three distinct gene clusters, suggesting amplification via tandem duplication during LTP family evolution (Figure 1A).

A distinct branching structure was observed in the phylogenetic tree of safflower LTP family members (Figure 1B). Combined with physicochemical properties (Table S1), motif analysis, and conserved domain analysis, members in the upper part of the phylogenetic tree were shorter, whereas those in the lower part were longer. Different branches harbored additional domains besides the basic LTP domain (Figure 1C,D), indicating that the family could be divided into several distinct subfamilies or evolutionary clades.

Promoter structure analysis revealed multiple hormone-responsive regions in the promoter regions of LTP family members, including regions responsive to auxin, MeJA, ABA, SA, and gibberellin. Stress-responsive regions for drought and low temperature, as well as defense-related regions for wound response and defense response, were also detected, implying that LTP genes are involved in responses to diverse stresses and hormone signals in plants (Figure 1E).

Variation in gene body length was detected among different family members, and no obvious clustering pattern related to these differences was observed in the phylogenetic tree (Figure 1F).

Based on gene family analysis, the disease-resistant candidate gene identified from GWAS, Chr2T10180100, was designated *CtLTP8*.

### 2.2. Proteome of Safflower EVs

Lipid transfer proteins (LTPs) are known to serve key roles in plant immunity and have been identified in extracellular vesicle (EV) proteomes from multiple plant species [20,27,34,52,53]. Based on their extracellular localization, CtLTP8 is predicted to reside in safflower EVs and mediate antifungal activity. To test this hypothesis, EVs were isolated from *B. cinerea*-infected safflower leaves and analyzed by proteomics.

Apoplastic washing fluid (AWF) was extracted from safflower leaves under *B. cinerea* infection. The purity of AWF was verified by electrolyte leakage assays [20]. Electrolyte leakage tests demonstrated that the increase in relative conductivity was of less than 1% after AWF extraction, indicating negligible intracellular contamination. Thus, the AWF was considered suitable for subsequent EV isolation (Table S2). EVs were then purified from safflower tissues by a two-step method involving ultracentrifugation and the Exosome Detection Ultrafast-isolation System (EXODUS) [11].

**Figure 2:**
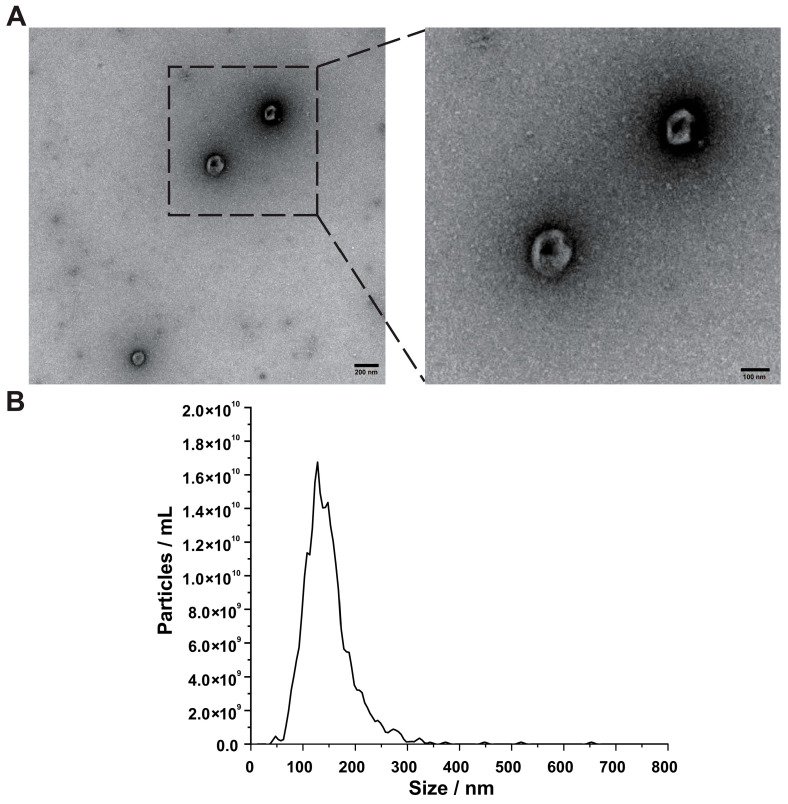
**Characterization of safflower EVs:** (**A**) transmission electron microscopy (TEM) observation of safflower EVs. Safflower EVs show a standard cup-shaped bilayer membrane structure with a clean background and no impurities. (**B**) Nanoparticle-tracking analysis (NTA) of safflower EVs. The particle size of safflower EVs is concentrated between 80 and 150 nm.

Typical bilayer cup-shaped structures of safflower EVs were observed by transmission electron microscopy (TEM), consistent with the basic morphology of plant AWF-derived EVs. Intact structures and low background impurities were detected, supporting the reliability of subsequent EV proteomic analysis (Figure 2A). Nanoparticle-tracking analysis (NTA) showed that safflower EVs were of 80–150 nm in diameter (Figure 2B). The above TEM and NTA characterizations were consistent with the standard morphology of plant AWF-derived EVs [21,28].

**Figure 3:**
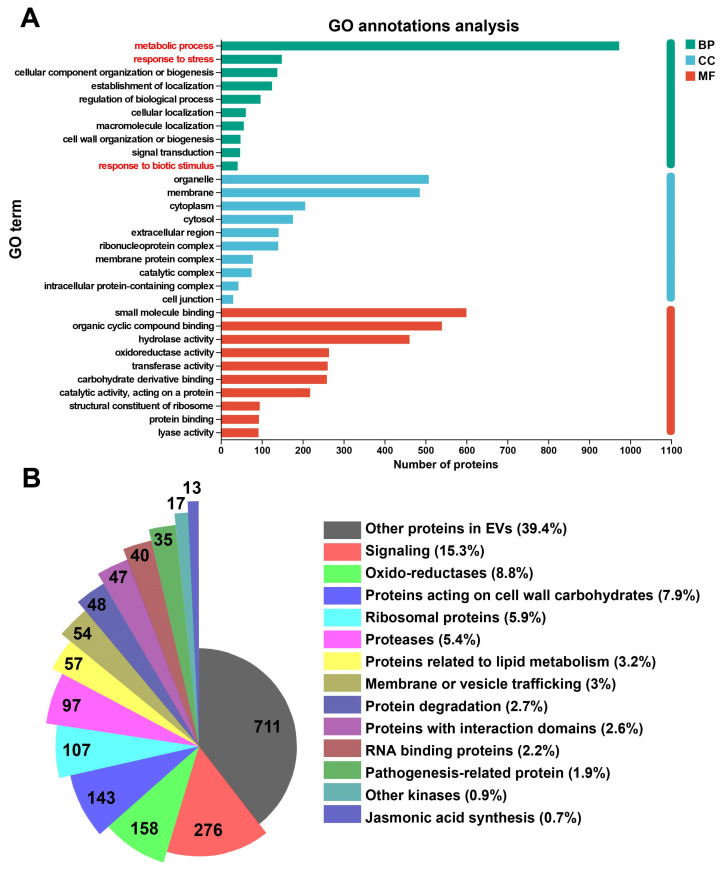
**Proteome of safflower EVs: **(**A**) GO annotation analysis of proteins derived from safflower leaf EVs. (**B**) Functional classification of proteins derived from safflower leaf EVs. The numbers in the chart indicate the counts of protein members in each category.

Further proteomic analysis of safflower leaf EVs was conducted. A total of 1803 EV proteins were identified. Gene Ontology (GO) analysis was performed for the identified proteome. The results showed that the proteome of safflower leaf EVs contained numerous plant defense-related proteins and proteins with catalytic activity, including proteins associated with terms such as “response to stress” and “response to biotic stimulus” (Figure 3A).

Protein sequences of safflower EVs were matched to the *Arabidopsis* database by homologous alignment. Based on their potential functions in EV biology, these proteins were classified into 14 categories: jasmonic acid synthesis; membrane or vesicle trafficking; other kinases; RNA binding proteins; pathogenesis-related protein; ribosomal proteins; protein degradation; proteins with interaction domains; related to lipid metabolism; oxido-reductases; signaling; proteases; proteins acting on cell wall carbohydrates; and other proteins in EVs (Figure 3B, Table S3).

Functional classification revealed a large number of “Proteins acting on cell wall carbohydrates” in safflower EVs. These proteins are associated with cell wall modification or degradation of cell walls from invading pathogens. Abundant “Oxido-reductases” and “Pathogenesis-related proteins” were also detected, which are closely related to plant defense responses. In addition, a large number of “Signaling” proteins were identified, reflecting the critical role of EVs as important mediators for signal perception and transduction [21,54].

Representative proteins from each category were selected and listed in Table 1. Detailed classification information for all proteins is provided in the supplementary table (Table S3). As shown in Table 1, safflower EVs contain multiple defense-related proteins, including pathogenesis-related protein 1 (PR1), CtTLP13, CtChi19 (Chitinase), lipid-transfer protein (CtLTP8), catalase, and germin-like protein. Among these, CtTLP13 has been clearly verified to exhibit antifungal activity both in vivo and in vitro [11].

In addition, common components of plant EV proteomes were detected in safflower EVs, including 14-3-3 proteins, heat shock proteins, ribosomal proteins, pectin methylesterase inhibitor (PMEI), calreticulin, Argonaute, and tetraspanin. Tetraspanin is a marker protein for plant EVs and is homologous to CD63, a characteristic marker protein for mammalian EVs [27,28,52,55]. These results indicate that safflower EVs contain numerous conserved proteins shared by plant EV proteomes, as well as abundant defense-related proteins.

A total of 18 lipid transfer proteins, including CtLTP8, were identified in safflower EVs, suggesting that lipid transfer proteins may exert important disease-resistant functions in safflower EVs.

### 2.3. CtLTP8 Is Localized in Safflower EVs

**Figure 4:**
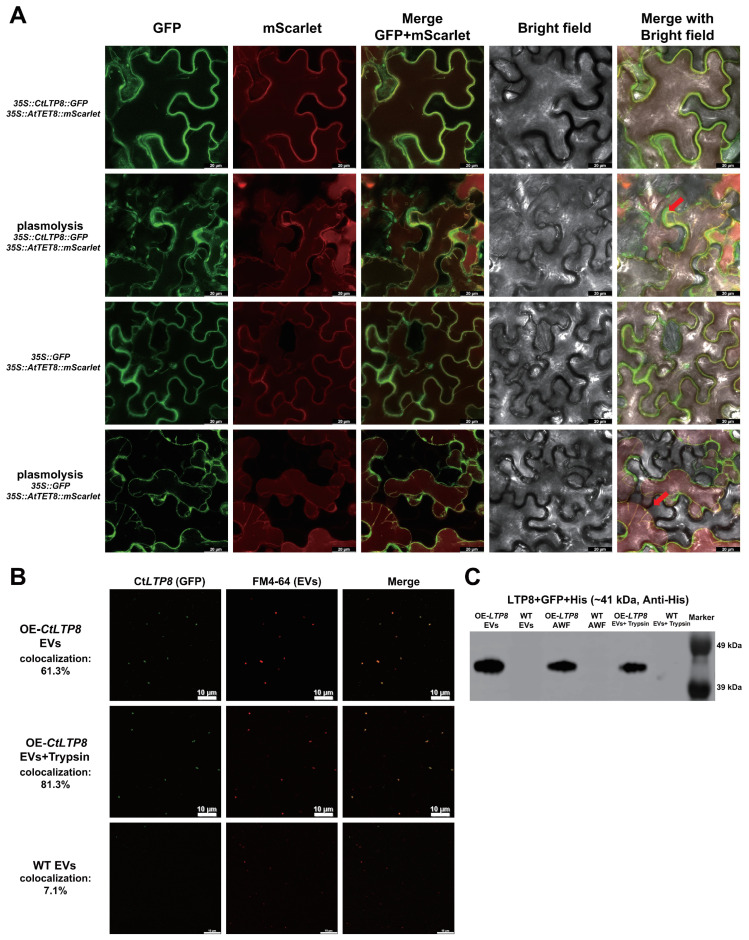
**Subcellular localization of CtLTP8 and its presence in EVs:** (**A**) transient expression in tobacco. After plasmolysis, CtLTP8 and AtTET8 were co-localized in the extracellular space and plasma membrane. Extracellular regions after plasmolysis are indicated by red arrows. GFP signals represent CtLTP13, and red mScarlet signals represent AtTET8. Scale bar: 20 μm. (**B**) Fluorescence co-localization assay. EVs from OE-*CtLTP8* transgenic plants showed green fluorescent signals due to the GFP tag, while EVs labeled with the red fluorescent dye FM4-64 showed red fluorescent signals. Co-localization of the two signals indicated that CtLTP8 is localized in EVs. After trypsin digestion, green signals of GFP-labeled CtLTP8 remained co-localized with red signals of FM4-64-labeled EVs, confirming that CtLTP8 is localized inside EVs rather than on the EV surface. Scale bar: 10 μm. (**C**) Western blot detection. Anti-His antibody detection showed that CtLTP8 was present in EVs and AWF, and CtLTP8 was still detected after trypsin digestion.

To verify the presence of CtLTP8 in safflower EVs, the *35S::CtLTP8-GFP-His* vector was constructed (Figure S2A) and verified by PCR (Figure S2B,C). Transgenic safflower plants overexpressing *CtLTP8* were generated via *Agrobacterium*-mediated pollen tube transformation, and T2-generation positive lines were identified by PCR (Figure S2D). The presence of CtLTP8 in safflower EVs was confirmed by fluorescence observation and Western blotting (Figure 4).

AtTET8 is a marker protein of *Arabidopsis* EVs homologous to the mammalian EV marker CD63, and is localized on the membrane of *Arabidopsis* EVs. The *35S::AtTET8-mScarlet* vector was constructed and co-expressed with *35S::CtLTP8-GFP-His* in tobacco via transient transformation, and the presence of CtLTP8 was investigated. The results showed that after plasmolysis, CtLTP8 and AtTET8 were co-localized in the extracellular space and plasma membrane in tobacco (Figure 4A).

To further confirm whether CtLTP8 is localized in safflower EVs, EVs were isolated from safflower plants overexpressing *CtLTP8* (OE-*CtLTP8*) and WT plants. GFP signals of the CtLTP8-GFP fusion protein were observed to co-localize with FM4-64-stained EV signals (red) in EVs from OE-*CtLTP8* plants under confocal laser scanning microscopy (CLSM) (Figure 4B).

Co-localization was maintained after trypsin digestion, indicating that CtLTP8 is localized inside safflower EVs rather than attached to the EV surface (Figure 4B). CtLTP8 was further verified by Western blotting (WB) in both EVs and AWF isolated from OE-*CtLTP8* transgenic plants. CtLTP8 was still detected in EVs from OE-*CtLTP8* transgenic plants after trypsin digestion (Figure 4C). The WB results were consistent with fluorescence results (Figure 4B), confirming that CtLTP8 is localized inside safflower EVs rather than on the EV surface. These results also validate the reliability of the safflower EV proteome.

### 2.4. Transgenic Verification Indicates That CtLTP8 Enhances Resistance to Botrytis cinerea in Safflower

**Figure 5:**
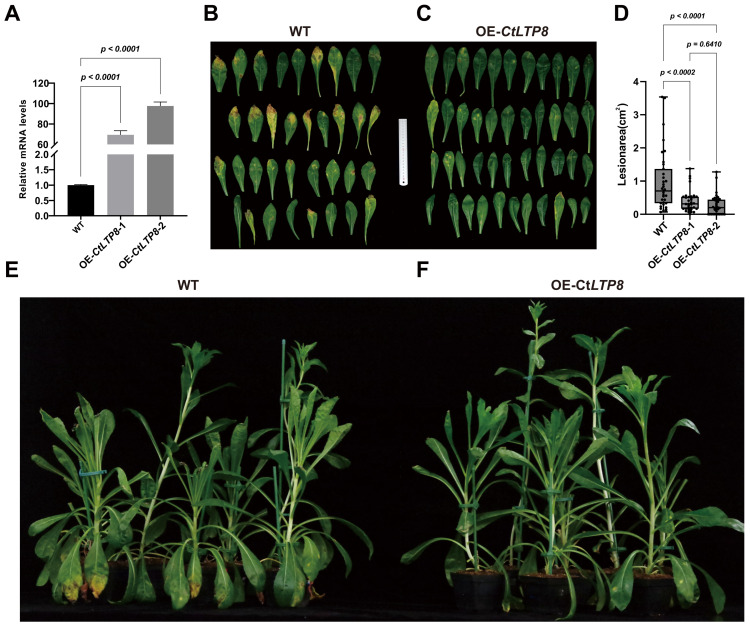
**OE-CtLTP8 enhances resistance to *Botrytis cinerea* in safflower:** (**A**) two high-expression OE-*CtLTP8* overexpression lines were obtained via screening. (**B**) Typical leaves of wild-type (WT) plants at 5 days post inoculation (dpi) with *B. cinerea* mycelia. (**C**) Typical leaves of OE-*CtLTP8* transgenic plants at 5 days post inoculation with *B. cinerea* mycelia. (**D**) Lesion areas were statistically analyzed using ImageJ at 5 dpi (n = 8 plants per group). Resistance to *B. cinerea* was significantly enhanced in both overexpression lines. (**E**) Typical WT plants at 5 days post inoculation with *B. cinerea* mycelia. (**F**) Typical OE-*CtLTP8* overexpression plants at 5 days post inoculation with *B. cinerea* mycelia.

Two high-expression OE-*CtLTP8* transgenic safflower lines were identified by reverse transcription quantitative real-time PCR (RT-qPCR). The relative expression levels of *CtLTP8* in these two lines were increased by 70-fold and 97-fold compared with those in the wild type (Figure 5A).

Two overexpression lines and wild-type plants were inoculated with *B. cinerea* mycelia. Lesion areas were compared (Figure 5B,C) and statistically analyzed using ImageJ (Version 1.53a). The results showed that resistance to *B. cinere* was significantly enhanced in both overexpression lines (Figure 5D). Moreover, comparison of typical infected plants indicated that OE-*CtLTP8* plants exhibited healthier phenotypes (Figure 5E,F).

In summary, these results confirm that CtLTP8 is localized in safflower EVs and that overexpression of *CtLTP8* can improve resistance to *B. cinere* in safflower.

## 3. Discussion

EVs represent ideal carriers and play a pivotal part in plant disease resistance. The transport of RNAs and proteins between hosts and pathogens is mediated by EVs, which are regarded as an important molecular basis for regulating the process of plant–microbe interactions [21,25,56,57]. Safflower EVs isolated in this study are bilayer membrane nanoparticles measuring 80–150 nm in diameter (Figure 2B,C), which conform to the typical morphological characteristics of plant EVs [21,54].

In plant–microbe interactions, an interactive and competitive system between plants and pathogens is formed by interspecies bidirectional transport mediated by EVs. Small RNAs (sRNAs) and mRNAs are delivered to invading fungi by plants via EVs to inhibit pathogen growth through cross-kingdom RNA interference (cross-kingdom RNAi). In *Arabidopsis*, mRNAs are transported into fungal cells via EVs and translated into functional proteins to block infection [13,19,28,29,58,59]. Conversely, EVs are secreted by diverse pathogenic fungi and oomycetes to promote infection. For instance, *Botrytis cinerea* delivers EVs via clathrin-mediated endocytosis and hijacks host AGO1 proteins to suppress immunity, while *Rhizoctonia solani* and *Phytophthora* species transport effector proteins via EVs to target plant immune pathways [22,24,60,61,62,63].

Critical roles in disease defense are exerted by plant EVs. The secretion of EVs is upregulated in *Arabidopsis*, rice and other plants under biotic stress. In addition, broad-spectrum antimicrobial activities against fungi and bacteria are exhibited by plant EVs, and EVs from *Arabidopsis* are capable of delaying the process of fungal infection [11,27,28,64,65,66]. Furthermore, EVs are involved in the transduction and cascade amplification of plant immune signals, and are recognized as essential components for the establishment of systemic acquired resistance (SAR). Bioactive substances such as RNAs and proteins carried by EVs are crucial for immune regulation [52].

The protein composition of EVs from infected safflower was analyzed and classified based on their biological functions in EVs (Figure 3, Table 1 and Table S3). A series of classic associated proteins were identified, including 14-3-3 proteins, lipid transfer proteins, heat shock proteins, pathogenesis-related proteins, and pectin methylesterase inhibitors (PMEIs) as representative components. These proteins are involved in core physiological processes such as EV biogenesis, signal transduction, and stress resistance. Tetraspanin, a homologous protein of the mammalian EV marker CD63, was also detected and can be utilized as a conserved marker for plant EVs (Figure 3, Table 1 and Table S3) [20,27,52,55,64,65]. Meanwhile, the plant apoplast contains numerous enzymes related to reactive oxygen species (ROS) and reactive nitrogen species (RNS), which defend against fungal invasion by inhibiting pathogens, triggering HR, reinforcing cell walls, mediating ROS–RNS crosstalk, and inducing systemic resistance [67]. This study further detected abundant oxidoreductases in safflower EVs, including peroxidases, catalase, peroxiredoxin, thioredoxin, and RNS-related enzymes such as nitrate reductase. It is therefore speculated that safflower EVs may enhance antifungal resistance through ROS/RNS pathways.

These results are consistent with previously published proteomic studies of EVs from multiple plant species. Defense-related proteins including PR family proteins, chitinases, and leucine-rich repeat (LRR) proteins are commonly identified in these studies. Moreover, such immune proteins are constitutively enriched in EVs from uninfected plants, indicating that EV-mediated protein transport is a conserved mechanism widespread in the plant kingdom [20,27,52,53,55,64,65,68]. In addition, previous studies have verified that EVs from both wild-type and transgenic safflower exhibit direct antifungal activity against *B. cinerea* in vitro [11]. The protein composition of safflower EVs was systematically characterized in this study, which further enriches the gene resources for safflower disease-resistant breeding and provides numerous key candidate genes for antifungal research in safflower.

Safflower LTPs are classified into the pathogenesis-related protein 14 (PR14) family, and a total of 77 members of the LTP gene family are identified (Figure 1). Plant LTPs exert critical functions in antifungal defense. LTPs in most plant species contain an N-terminal signal peptide and are secreted into the extracellular space to perform diverse physiological functions. The results of this study further extend this conclusion and verify that CtLTP8 in safflower is distributed in both AWF and EVs (Figure 4B,C). It is therefore suggested that plant LTPs may execute multiple physiological functions via EVs.

With regard to the direct inhibition of fungal growth, significant in vitro antifungal effects of LTPs from multiple plant species are documented. For example, *Arabidopsis* LTP4.4 effectively suppresses the growth of *Fusarium graminearum* [69], and LTPs from *Gossypium hirsutum* exhibit distinct inhibitory effects on the proliferation of diverse fungi [70]. BrLTP2.1 from *Brassica napus*, a nectar protein, displays potent broad-spectrum antifungal activity against necrotrophic fungi [48]. It is speculated in this study that once delivered into fungal cells via EVs, CtLTP8 may exert direct antifungal activity through two major mechanisms. First, by virtue of its lipid-binding capacity, CtLTP8 may extract lipids from the fungal cell membrane, thereby increasing membrane permeability and impairing its structural integrity [33]. Second, the highly conserved cationic residues on the surface of CtLTP8 may interact with negatively charged domains on fungal membranes, leading to membrane destabilization [71]. Notably, even LTP proteins with impaired lipid-binding activity may retain partial antifungal activity through their cationic residues. For example, rice LTP110 may still inhibit Magnaporthe oryzae after site-directed mutagenesis disrupts its lipid-binding structure [72]. Accordingly, it is inferred in this study that safflower CtLTP8 may confer disease resistance by directly disrupting fungal cell membranes. Transgenic safflower plants are constructed in this study (Figure 5A), and enhanced resistance to *B. cinerea* conferred by *CtLTP8* overexpression is confirmed through fungal inoculation assays (Figure 5B–F).

At the level of physical defense on the plant surface, antifungal barrier functions are strengthened by LTPs through the regulation of cuticular wax accumulation [73]. The plant cuticle serves as the first physical defense line against pathogen invasion, and its wax components are composed of long-chain fatty acids and their derivatives. LTPs are directly involved in the transport of cutin monomers and waxes [74]. *Arabidopsis* LTPg1, LTPg2 and LTPg6 play key roles in cuticular lipid accumulation. Loss of function of LTPg1 leads to disordered and diffuse cuticular structures and significantly increases plant susceptibility to *Alternaria brassicicola* [36,73,75]. *Brassica rapa* BraLTP1 directly enhances plant antifungal capacity by participating in epidermal wax deposition [76]. In addition, *Arabidopsis* LTP2 exerts minor effects on cuticular components but specifically maintains the adhesive integrity between the cuticle and cell wall, and its expression is induced by *B. cinerea*, further confirming the indispensable role of LTPs in surface defense [77,78]. Meanwhile, *Arabidopsis* LTPg1, LTPg2, LTPg5 and LTPg6 participate in pre-invasion defense against non-host powdery mildew fungi (e.g., *Blumeria graminis* f. sp. *hordei*), and loss of function of these LTPs elevates the penetration rate of pathogens into epidermal cell walls [79].

Antifungal immunity in plants is regulated by some LTPs through hormone signaling pathways [80]. Potato StLTP10 interacts with the ABA receptor PYL4 to induce stomatal closure and resist *Phytophthora* infestans invasion. Tobacco LTP1 and grapevine VvLTP4 activate antifungal defense responses by binding jasmonic acid (JA) [80,81,82]. Meanwhile, a subset of LTPs negatively regulate fungal immunity to maintain defense homeostasis. For example, wheat TaDIR1-2 inhibits resistance to stripe rust, and *Arabidopsis* ACD11 suppresses programmed cell death by transporting ceramide-1-phosphate to avoid excessive immune activation [50,51,83]. These diverse regulatory modes collectively form an important regulatory network of LTPs in plant antifungal defense.

Plants exhibit distinct cell-type specificity and spatial defense microdomains during fungal resistance [84], suggesting that the antifungal function of *LTP8* is likely not exerted uniformly across all tissues, but rather dominantly expressed in cells near infection sites to participate in local immunity. Meanwhile, it is speculated that EVs can act as extracellular transporters for defense molecules such as LTP8, mediating intercellular signaling and antimicrobial cargo delivery, thereby precisely constructing spatial defense barriers.

## 4. Materials and Methods

### 4.1. Plant and Fungal Materials

Safflower seeds of cultivar Yunhong-7 were used as experimental materials. Single seeds were sown in pots, and the cultivation substrate was prepared by mixing vermiculite, perlite and nutrient soil at a volume ratio of 1:1:1. Plants were cultivated under long-day conditions (16 h light/8 h dark, 22–26 °C). Plants at the 6–8-leaf stage (approximately 3–5 weeks old) were used for *Botrytis cinerea* inoculation, followed by EV isolation. Safflower plants for genetic transformation were cultivated for 6–8 weeks until the flowering stage.

*B. cinerea* strain B05.10 (NCBI Taxonomy ID: 332648) was inoculated on potato dextrose agar (PDA (Solarbio, Beijing, China)) plates and cultured at 28 °C for 3–4 weeks. Mycelial blocks of uniform size were obtained using a punch, attached to the surface of safflower leaves for fungal stress inoculation, and incubated under dark and humid conditions for 24 h. Subsequently, the plants were returned to the aforementioned long-day conditions for further cultivation.

### 4.2. Linkage Disequilibrium Block Analysis

Linkage disequilibrium (LD) block analysis was performed for significantly associated single-nucleotide polymorphisms (SNPs) within the 300 kb flanking region of the target SNP locus using LDBlockShow [85]. Functional annotation and localization of significant SNP loci were conducted using the ANNOVAR (Version 2025Mar21) software combined with the reference genome annotation file [1,86].

### 4.3. Gene Family Analysis

Sequences of the *Arabidopsis* LTP gene family were downloaded from the TAIR database. Homologous sequences were screened by alignment against the safflower genome via the BLASTP program. The safflower genome was searched using HMMer V3.4 software (http://www.hmmer.org/) with hidden Markov model profile files PF14368 and PF00234 for conserved LTP domains from the Pfam database (Protein families database) [87].

Candidate sequences of safflower LTP family members were obtained by integrating the results from BLASTP (Veision 2.9.0) and HMMer searches. Conserved domain analysis of the candidate sequences was subsequently performed using the NCBI Conserved Domain Database (CDD) and InterPro database. Sequences with incomplete conserved domains or excessively short lengths were excluded [88]. Promoter sequences 2000 bp upstream of the initiation codon of LTP family members were extracted, and cis-acting regulatory elements were analyzed using the PlantCARE online tool (https://bioinformatics.psb.ugent.be/webtools/plantcare/html/, accessed on 10 May 2026) [89].

### 4.4. Extraction of Safflower AWF and Electrolyte Leakage Assay

Apoplastic washing fluid (AWF) was extracted from wild-type safflower leaves and leaves treated with *B. cinerea* for 36 h, based on the optimized protocol for *Arabidopsis* AWF isolation [11,64]. The degree of cellular contamination in AWF was detected via electrolyte leakage assays according to the method reported by Campos [90]. The electrical conductivity of safflower leaves was measured before and after AWF extraction, and the total conductivity was determined after incubation in a water bath at 80 °C for 4 h. The relative electrolyte leakage rate was calculated subsequently. The results showed that the relative increase in leaf conductivity induced by AWF extraction was of less than 1%, indicating intact leaf cell membranes and negligible cellular contamination of AWF, which was qualified for extracellular vesicle (EV) isolation. The experiment was repeated four times with consistent trends.

### 4.5. Isolation of Safflower EVs

Safflower EVs were isolated with reference to the method for *Arabidopsis* EV separation, and a two-step procedure consisting of crude extraction via ultracentrifugation and subsequent purification via Exosome Detection Ultrafast-isolation System (EXODUS) was performed in accordance with the optimized protocol established in previous studies [11]. Purified AWF was ultracentrifuged at 100,000× *g* for 1 h at 4 °C. The supernatant was discarded, and the pellet was resuspended in phosphate-buffered saline (PBS). Further purification of the crude extract was conducted using an Exosome Detection Ultrafast-isolation System (EXODUS) purifier (Model H-600, Huixin Life Technology Co., Ltd., Shenzhen, China) based on the principles of negative pressure oscillation and membrane vibration mediated by dual-coupled harmonic oscillators [91]. Finally, the purified EVs were resuspended in 100 μL PBS to obtain high-purity safflower EVs.

### 4.6. TEM and NTA Characterization

Ten microliters of safflower EVs resuspended in PBS were dropped onto 200-mesh carbon-coated copper grids and incubated at room temperature for 10 min. Negative staining was performed with 2% (*w*/*v*) phosphotungstic acid for 3 min. Residual liquid was absorbed with filter paper, and the morphological structure of EVs was observed using a transmission electron microscope (TEM, JEM1400, JEOL Ltd., Tokyo, Japan).

Ten microliters of purified EV samples was diluted with 1× PBS buffer. A nanoparticle tracking analysis (NTA) system (Zetaview-PMX120-Z, Particle Metrix GmbH, Meerbusch, Germany) was calibrated with 100 nm polystyrene microspheres. NTA data collection and analysis were completed at 11 detection sites under temperatures ranging from 23 °C to 30 °C.

### 4.7. Proteomic Analysis of Safflower EVs

Mass spectrometry detection of the safflower EV proteome was performed using data-independent acquisition (DIA) [92]. Protein quantification of the isolated EVs was conducted via the BCA assay. Peptide preparation was carried out using a commercial enzymatic hydrolysis kit (Majorbio Bio-Pharm Technology Co., Ltd., Shanghai, China). Enzymatic hydrolysis was performed at a constant temperature of 37 °C for 16 h to ensure complete degradation of proteins into peptides. The enzymatic hydrolysate was desalted using a C18 solid-phase extraction column to eliminate impurity interference. The desalted peptide eluate was freeze-dried using a vacuum concentrator and then redissolved in 0.1% formic acid (FA) aqueous solution. Peptide quantification was performed via ultraviolet spectrophotometry using a micro ultraviolet–visible spectrophotometer (NanoDrop One, Thermo Fisher Scientific Inc., Wilmington, DE, USA). Finally, 20 μg of peptides was dissolved in mass spectrometry loading buffer.

Equal amounts of peptides were dissolved in UPLC loading buffer and separated via high-pH liquid chromatography using a reversed-phase C18 column. The chromatographic system was a Thermo Scientific Vanquish Flex UHPLC system equipped with an Acquity UPLC BEH C18 Column (1.7 µm, 2.1 mm × 150 mm, Waters Corp., Milford, MA, USA). Equal amounts of peptides from different fractions separated by UPLC were dissolved in mass spectrometry loading buffer for DIA detection and analysis.

Peptide separation was performed using a Vanquish Neo (Thermo Fisher Scientific Inc.) chromatograph with a uPAC High Throughput column (75 μm × 5.5 cm, Thermo Fisher Scientific Inc., Sunnyvale, CA, USA). Mobile phase A consisted of water supplemented with 2% acetonitrile and 0.1% formic acid, and mobile phase B consisted of water supplemented with 80% acetonitrile and 0.1% formic acid. The chromatographic run time was set to 8 min. Data acquisition was conducted using Compass HyStar software (Version 4.1, Bruker Corp., Ettlingen, Germany).

Samples separated via nano-high performance liquid chromatography were subjected to mass spectrometry analysis using a timsTOF Ultra2 mass spectrometer (Bruker Corp., Ettlingen, Germany). The mass spectrometer was operated in DIA mode with positive ion detection, and the ion source voltage was set to 4.5 kV. The mass spectrometry scan range was 100–1700 *m*/*z*. The spectral library was imported into Spectronaut™ 19 software for database search analysis of the sample DIA data. Parameter settings were as follows: protein FDR ≤ 0.01; peptide FDR ≤ 0.01; peptide confidence ≥ 99%; XIC width ≤ 75 ppm. Protein quantification was performed using the MaxLFQ method. All data were uploaded to the Majorbio Cloud Platform (https://v.majorbio.com/user-center/login?redirect_url=https://v.majorbio.com/, accessed on 2 September 2025) for bioinformatic analysis [93].

### 4.8. GO Annotation Analysis

Gene Ontology (GO) annotation was completed using InterProScan (Version 5.76-107.0) software based on the safflower whole-genome protein sequence database (https://www.scuec.edu.cn/safflower/, accessed on 1 September 2025). A species-specific annotation library for safflower was constructed using the R package AnnotationForge. GO analysis of all identified proteins was performed using the clusterProfiler package [94]. The Benjamini–Hochberg algorithm was applied for *p*-value correction, with 0.05 set as the screening threshold. The top 10 terms ranked by protein count in each category, namely biological process (BP), cellular component (CC), and molecular function (MF), were presented.

### 4.9. Vector Construction

The CDS sequence of CtLTP8 (with the stop codon removed) flanked by homologous arms for Nco I and Spe I double digestion, as well as the AtTET8-mScarlet fragment (with the stop codon of AtTET8 removed), were obtained via PCR amplification. These fragments were ligated into the linearized pCAMBIA1302 vector (digested with Nco I and Spe I) via homologous recombination. The recombinant vectors *pCAMBIA1302-p35S::CtLTP8-GFP-His* and *pCAMBIA1302-p35S::AtTET8-mScarlet* were finally generated.

The recombinant vectors were transformed into Escherichia coli cells, and positive colonies were screened via PCR. Subsequently, the vectors were transferred into *Agrobacterium* cells. Positive colonies were verified by sequencing and preserved for subsequent use (primers are listed in Table S4).

### 4.10. Transient Expression in Tobacco

The subcellular co-localization analysis of CtLTP8 and AtTET8 was performed using an optimized transient expression system in *Nicotiana benthamiana* based on the method reported by Wydro [95]. *Agrobacterium tumefaciens* GV3101 harboring the recombinant vectors was cultured to an OD_600_ of 0.5–0.6. Bacterial cells were collected by centrifugation and resuspended in infiltration buffer (10 mM MgCl_2_, 10 mM MES, 150 μM acetosyringone), and the bacterial concentration was adjusted to an OD_600_ of 0.8.

The bacterial suspension was injected into the abaxial epidermis of young leaves from 4–5-week-old *N. benthamiana* plants using a needleless syringe. After 12 h of dark incubation, the plants were transferred to standard growth conditions (12 h light/12 h dark, 22 °C) and cultured for 2 days. The abaxial epidermal cells were observed using a confocal laser scanning microscope (Stellaris 5, Leica Microsystems GmbH, Wetzlar, Germany). Plasmolysis was induced by immersing the leaf abaxial epidermis in 0.4 M mannitol solution for 10 min prior to observation.

GFP fluorescence was excited by a 488 nm laser, and emission signals were collected at 500–550 nm. mScarlet fluorescence was excited by a 555 nm laser, and emission signals were collected at 595–630 nm.

### 4.11. Safflower Genetic Transformation

Genetic transformation of safflower was conducted according to previously reported methods [11,96]. Safflower plants at the early flowering stage (8–10 weeks old) were selected as recipients. *Agrobacterium tumefaciens* GV3101 harboring the CtLTP8 recombinant vector was cultured to an OD_600_ of 0.8. Bacterial cells were collected by centrifugation and resuspended in infiltration buffer supplemented with 5% sucrose and 0.02% Silwet-L77.

Floral buds and filaments were immersed in the buffer for no less than 1 min on the day of flowering, and inoculation was performed continuously for 2 days. The inoculated plants were cultivated under long-day conditions (16 h light/8 h dark, 22 °C) until T_0_ generation seeds were harvested. T_0_ seeds were sown and cultured under the same conditions. Positive transgenic plants were screened by PCR and propagated to homozygous T_2_ generation lines. The expression level of the target gene was detected by reverse transcription quantitative real-time PCR (RT-qPCR) (primers are listed in Table S4).

### 4.12. Trypsin Treatment of EVs

To determine whether CtLTP8 is localized inside or outside EVs, the experiment was performed following the method reported by Huang [27]. EVs were incubated in phosphate-buffered saline (PBS) containing 10 mg/mL trypsin at 37 °C for 30 min. Fluorescence signal images were subsequently observed and collected using a confocal laser scanning microscope (CLSM, Stellaris 5, Leica Microsystems, Wetzlar, Germany).

Fluorescence detection parameters were set as follows: green fluorescent protein (GFP) was excited by a 488 nm laser, and green fluorescence signals were collected at 500–550 nm; FM4-64 red fluorescence was excited by a 555 nm laser, and red fluorescence signals were collected at 580–650 nm. The co-localization characteristics of FM4-64 and GFP were analyzed using the co-localization function of the Leica Application Suite X (LAS X, v. 4.8.1.29271) software.

### 4.13. Western Blot Analysis

A His-tag monoclonal antibody (ABclonal Biotechnology Co., Ltd., Wuhan, China) was used to detect His-tagged CtLTP8 protein in the samples. *Apoplastic* washing fluid (AWF) and EVs isolated from *CtLTP8*-overexpressing transgenic plants (OE-*CtLTP8*) and wild-type (WT) plants were analyzed to clarify the localization characteristics of the CtLTP8 protein. A protein loading volume of 20 μg was applied for each sample. Positive signals were captured and analyzed using a fluorescence imaging system (LI-COR Biosciences, Lincoln, Lincoln, NE, USA).

## 5. Conclusions

Previously published GWAS data for disease resistance in safflower were reanalyzed, and the candidate disease-resistant gene *CtLTP8* was screened. Genome-wide analysis of the safflower LTP gene family was performed, and the chromosomal localization, phylogenetic characteristics, and stress-related promoter elements of 77 family members were identified. A method for the isolation and purification of apoplastic EVs in safflower was established, and typical bilayer membrane EVs with a diameter of 80–150 nm were obtained. The proteome of these EVs contained a variety of defense-related proteins and conserved markers, and 18 lipid transfer proteins including CtLTP8 were identified. Molecular experiments verified that CtLTP8 is localized inside safflower EVs, and overexpression of this gene significantly enhanced resistance to *B. cinerea* in safflower. This study clarified the disease-resistant mechanism of CtLTP8 mediated by EVs, enriched the disease-resistant gene resources of safflower, and provided a theoretical basis and candidate genes for the molecular breeding of disease-resistant safflower.

In this study, we obtained proteomic profiles of safflower EVs. However, due to technical difficulty and high cost of EV isolation, only *B. cinerea*-infected samples were analyzed without an uninfected control to reveal proteomic changes under fungal stress. Future studies may include more treatment groups to identify EV proteins responsive to fungal infection. In addition, the antifungal mechanism of CtLTP8 and its role in SAR signaling deserve further investigation, and this gene can be used in disease-resistant breeding of other economic crops. The specific antifungal mechanism of CtLTP8, its selective sorting and loading into EVs, and whether it functions alone or with other EV proteins are key biological questions in LTP-mediated antifungal immunity, which warrant further exploration. Meanwhile, whether CtLTP8 also confers resistance against other fungal diseases in safflower, such as those caused by *Alternaria* spp. and *Fusarium oxysporum*, represents an excellent direction for future research.

In summary, as a functional gene localized in EVs that can significantly enhance the resistance of safflower to *B. cinerea*, CtLTP8 is an ideal candidate gene for the molecular breeding of antifungal safflower, and can provide core genetic elements and breeding strategies for the cultivation of new disease-resistant safflower varieties. The proteomic dataset of safflower EVs constructed in this study systematically reveals the components of defense-related proteins in safflower EVs. This dataset not only fills the research gap of disease-resistant proteins in safflower EVs, but also lays an important data foundation for the subsequent analysis of EV-mediated immune mechanisms in safflower and the mining of more disease-resistant functional genes. It holds important reference value for promoting the basic research and breeding application of safflower disease resistance.

## Figures and Tables

**Table 1:** Representative proteins in each subgroup of EV proteins.

Accession_id	Classification	Description
CtAH01T0046200.1	Jasmonic acid synthesis	allene oxide cyclase
CtAH11T0079800.1	Jasmonic acid synthesis	allene oxide cyclase
CtAH05T0004100.1	Jasmonic acid synthesis	oxophytodienoate-reductase
CtAH02T0063300.1	Other kinases	arabinose kinase
CtAH05T0271500.1	Other kinases	kinase-like protein
CtAH12T0157000.1	Other kinases	phosphoglycerate kinase
CtAH01T0094700.1	RNA binding proteins	DEA(D/H)-box RNA helicase family protein
CtAH06T0018200.1	RNA binding proteins	RNA-binding (RRM/RBD/RNP motifs) family protein
CtAH08T0256000.1	RNA binding proteins	Argonaute family protein
CtAH05T0085700.1	Pathogenesis-related protein	PR-1
CtAH08T0272400.1	Pathogenesis-related protein	CtTLP13
CtAH05T0143300.1	Pathogenesis-related protein	CtLTP8
CtAH05T0143300.1	Proteins with interaction domains	CtPMEI13
CtAH01T0173500.1	Proteins with interaction domains	Mannose-binding lectin superfamily protein
CtAH07T0097700.1	Proteins with interaction domains	Kunitz family trypsin and protease inhibitor protein
CtAH09T0004700.1	Protein degradation	20S proteasome alpha subunit
CtAH09T0047900.1	Protein degradation	26S proteasome regulatory subunit Rpn
CtAH03T0012900.1	Protein degradation	ubiquitin family protein
CtAH04T0066300.1	Membrane or vesicle trafficking	secretion-associated RAS super family
CtAH07T0204100.1	Membrane or vesicle trafficking	vaculolar sorting receptor
CtAH10T0078000.1	Membrane or vesicle trafficking	vesicle-associated membrane protein
CtAH06T0252200.1	Related to lipid metabolism	GDSL-motif lipase
CtAH10T0072000.1	Related to lipid metabolism	Thioesterase/thiol ester dehydrase-isomerase
CtAH05T0213100.1	Related to lipid metabolism	Phospholipase A2 family protein
CtAH05T0104300.1	Proteases	Papain family cysteine protease
CtAH06T0297800.1	Proteases	Subtilisin-like serine endopeptidase family protein
CtAH12T0067000.1	Proteases	aspartic proteinase
CtAH05T0111000.1	Ribosomal proteins	Ribosomal protein L30/L7 family protein
CtAH06T0050100.1	Ribosomal proteins	60S acidic ribosomal protein family
CtAH07T0246000.1	Ribosomal proteins	Ribosomal protein L2 family
CtAH02T0024100.1	Proteins acting on cell wall carbohydrates	Pectin lyase-like superfamily protein
CtAH03T0152800.1	Proteins acting on cell wall carbohydrates	beta glucosidase
CtAH06T0018500.1	Proteins acting on cell wall carbohydrates	beta-1,3-glucanase
CtAH01T0051400.1	Oxido-reductases	laccase
CtAH10T0229700.1	Oxido-reductases	thioredoxin
CtAH05T0076600.1	Oxido-reductases	Peroxidase superfamily protein
CtAH01T0054500.1	Signaling	Leucine-rich receptor-like protein kinase family protein
CtAH12T0142600.1	Signaling	tetraspanin
CtAH06T0243300.1	Signaling	14-3-3 PROTEIN
CtAH01T0089600.1	Other proteins in EVs	actin
CtAH06T0078300.1	Other proteins in EVs	heat shock protein 60
CtAH07T0221500.1	Other proteins in EVs	vacuolar proton ATPase

## Data Availability

The original contributions presented in this study are included in this article. Further inquiries can be directed to the corresponding author.

## Supplementary Materials

The supporting information can be downloaded at: https://www.mdpi.com/article/10.3390/plants15101527/s1.

## Abbreviations

The following abbreviations are used in this manuscript:

AWF	Apoplastic Washing Fluid
CLSM	Confocal Laser Scanning Microscopy
DIA	Data-Independent Acquisition
dpi	Days Post Inoculation
EV	Extracellular Vesicles
GO	Gene Ontology
GWAS	Genome-Wide Association Study
LD	Linkage Disequilibrium
LTP	Lipid Transfer Protein
NTA	Nanoparticle-Tracking Analysis
PMEI	Pectin Methylesterase Inhibitor
PR	Pathogenesis-Related
SAR	Systemic Acquired Resistance
TEM	Transmission Electron Microscopy
