# Supplementary figures and images for: Localization and Functional Analysis of CtLTP8, an Extracellular Vesicle Protein That Enhances Resistance to Botrytis cinerea in Safflower
Source: Plants (Basel). 2026 May 16;15(10):1527. doi: 10.3390/plants15101527 (PMC13210472; doi:10.3390/plants15101527)

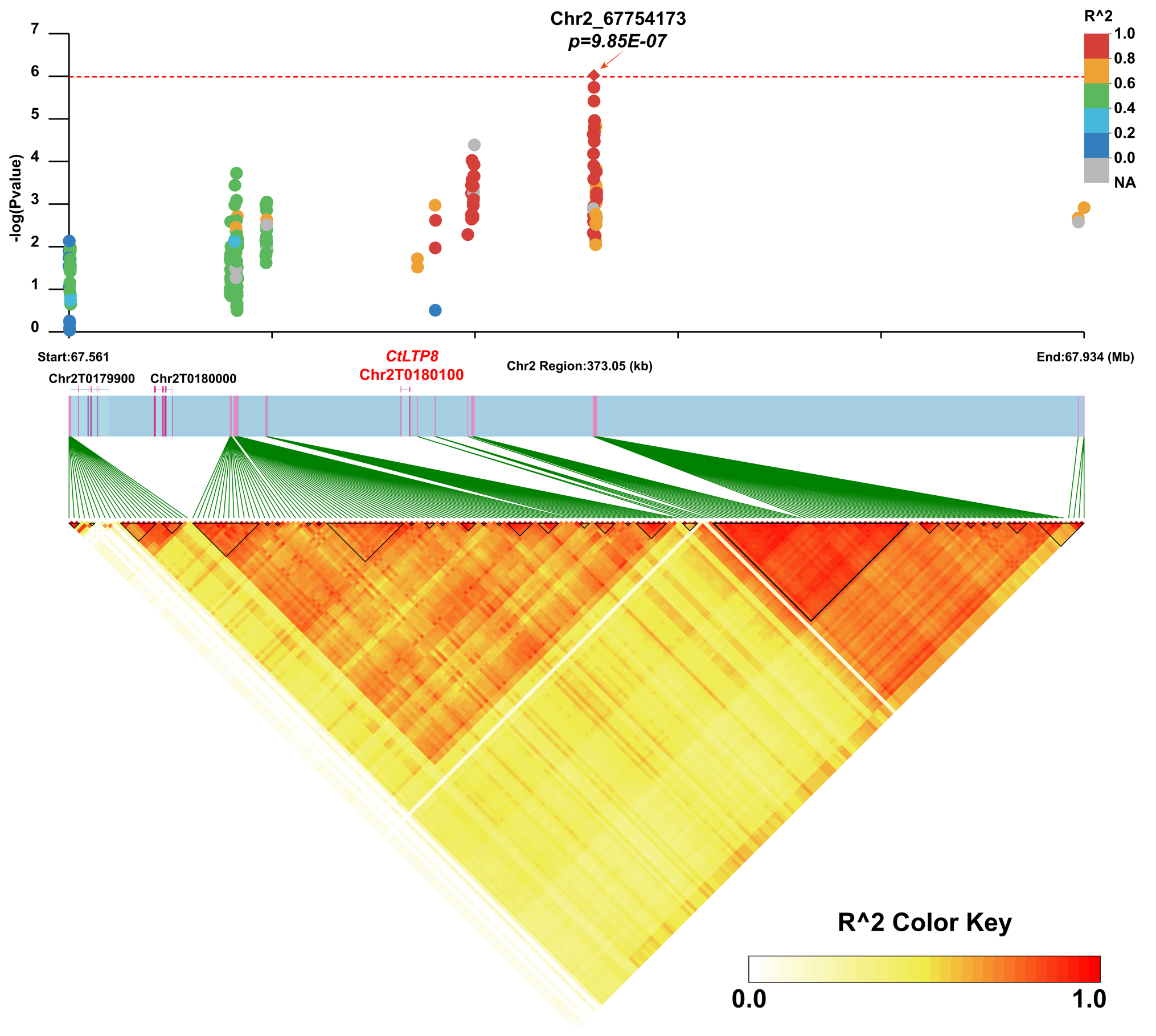

Supplement: Supplementary file 1 [file plants-15-01527-s001.zip › Supplementary Figure S1. Linkage disequilibrium block analysis of SNP loci.pdf]

**A**

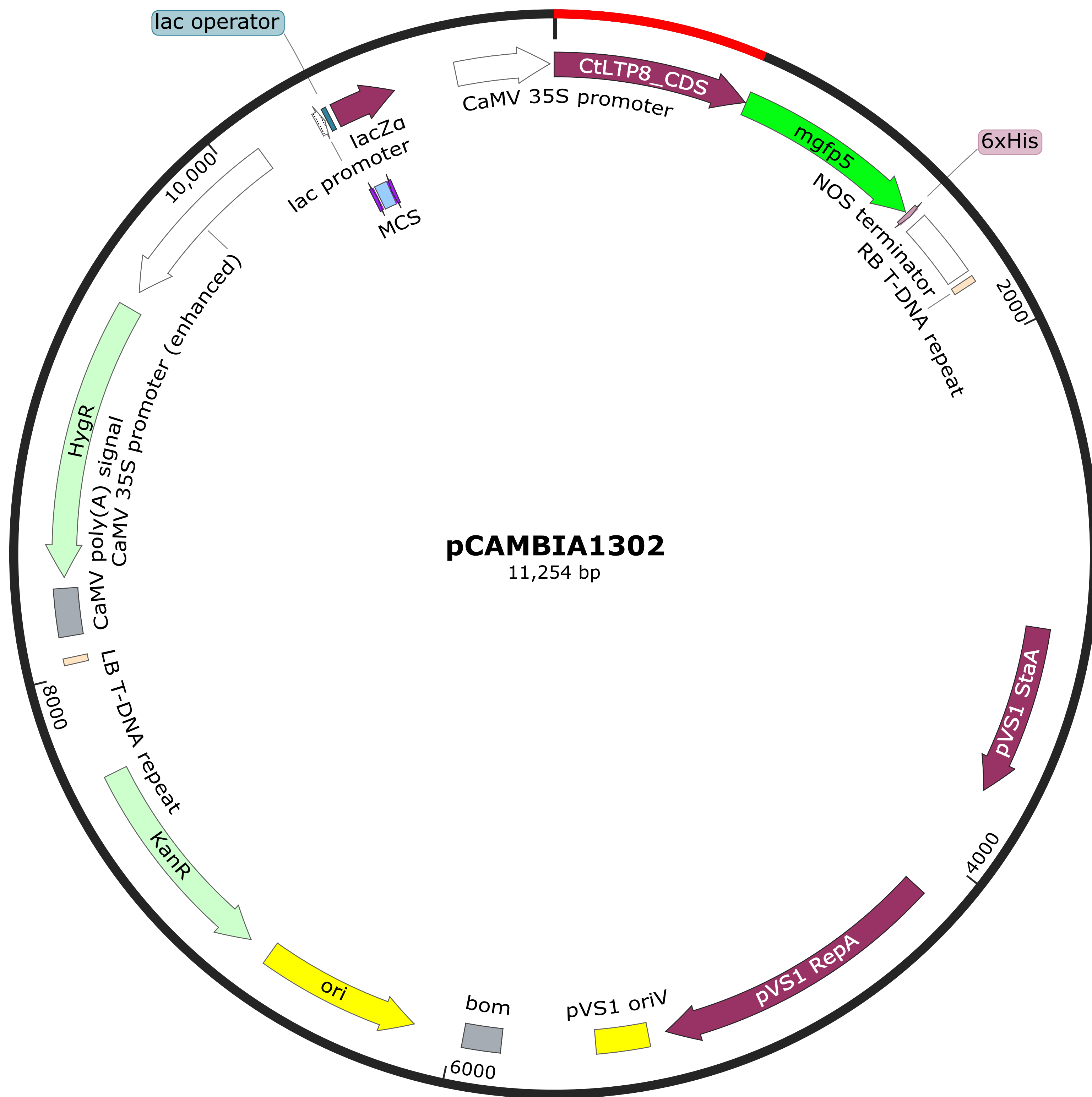

**B**

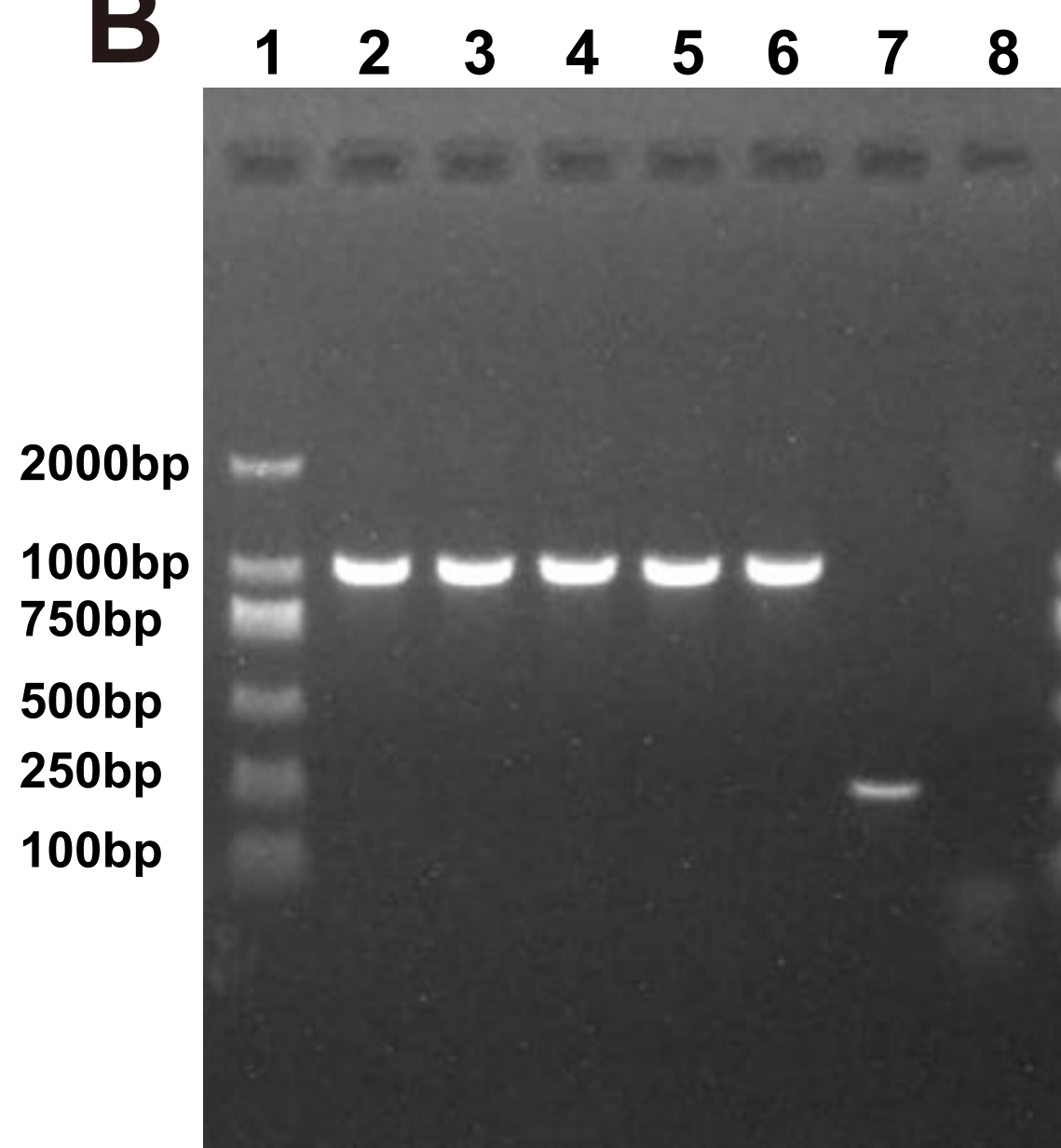

**C**

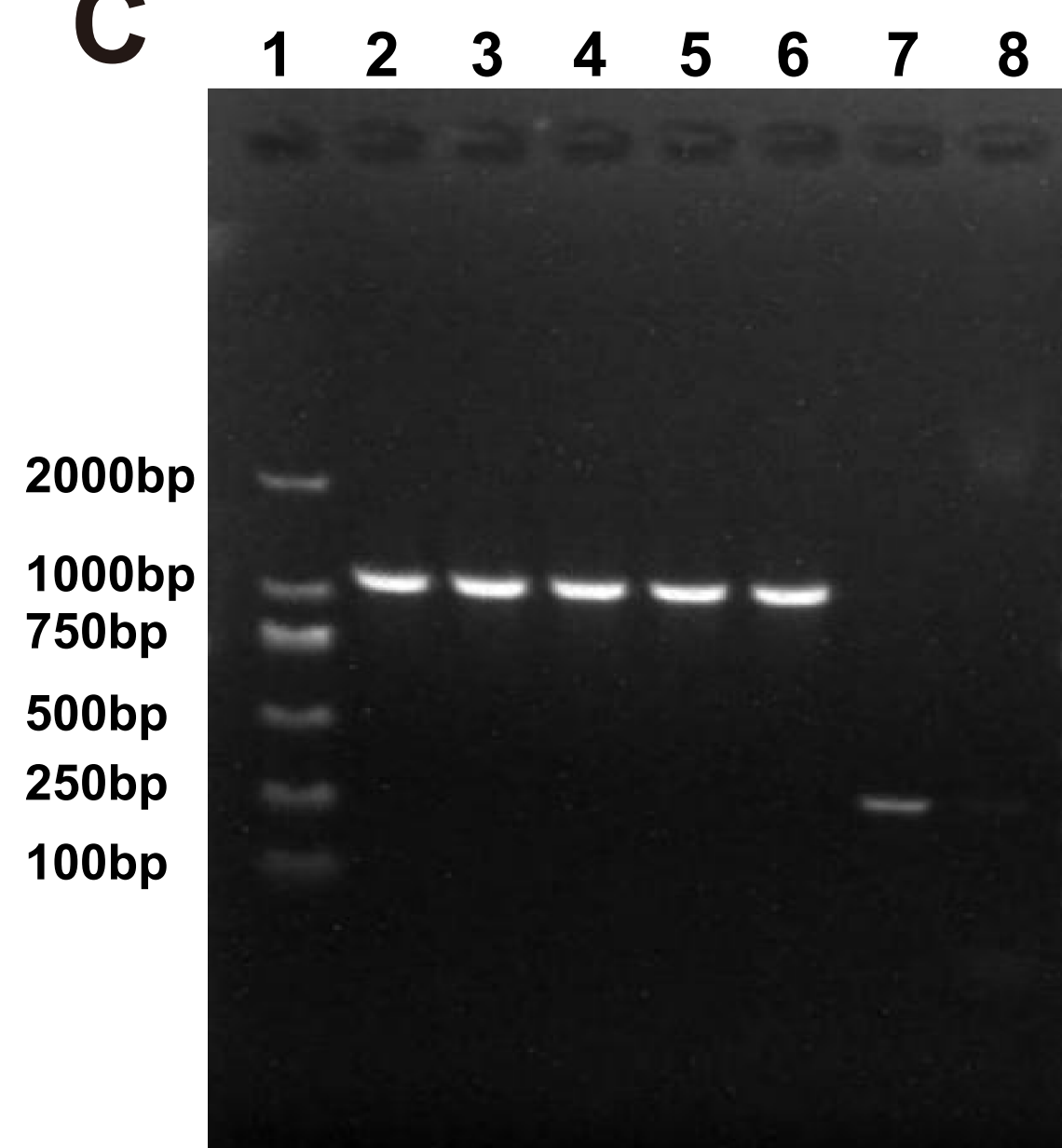

**D**

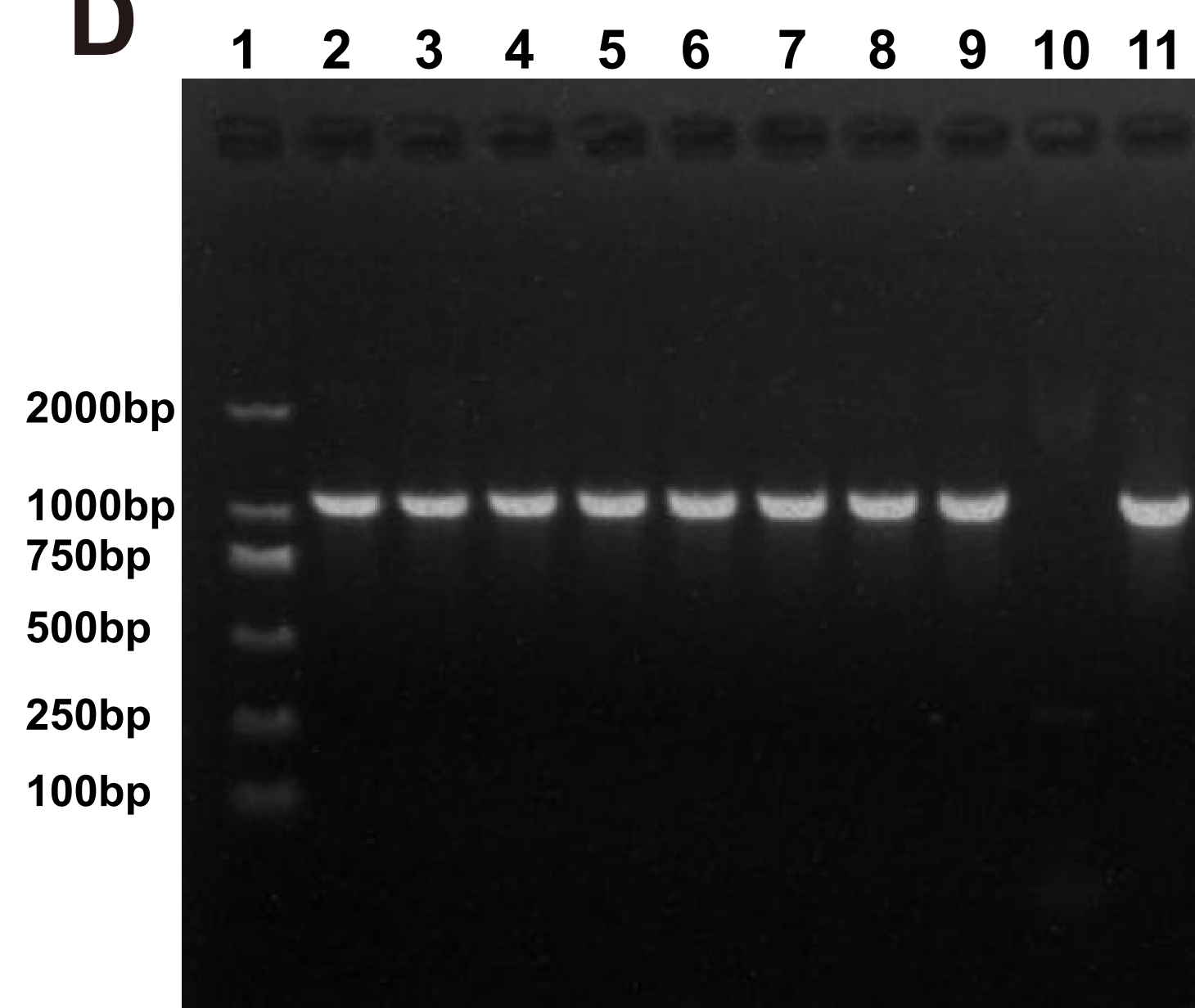

Supplement: Supplementary file 1 [file plants-15-01527-s001.zip › Supplementary Figure S2. Construction of the safflower CtLTP8 transgenic vector and screening of positive transgenic plants.pdf]
